# A Near-Infrared Cell Tracker Reagent for Multiscopic In Vivo Imaging and Quantification of Leukocyte Immune Responses

**DOI:** 10.1371/journal.pone.0001075

**Published:** 2007-10-24

**Authors:** Filip K. Swirski, Cedric R. Berger, Jose-Luiz Figueiredo, Thorsten R. Mempel, Ulrich H. von Andrian, Mikael J. Pittet, Ralph Weissleder

**Affiliations:** 1 Center for Molecular Imaging Research, Massachusetts General Hospital and Harvard Medical School, Charlestown, Massachusetts, United States of America; 2 Center for Immunology and Inflammatory Diseases, Massachusetts General Hospital, Charlestown, Massachusetts, United States of America; 3 CBR Institute for Biomedical Research and Department of Pathology, Harvard Medical School, Boston, Massachusetts, United States of America; 4 Center for Systems Biology, Massachusetts General Hospital and Harvard Medical School, Boston, Massachusetts, United States of America; Centre de Recherche Public-Santé, Luxembourg

## Abstract

The complexity of the tumor microenvironment necessitates that cell behavior is studied in a broad, multi-scale context. Although tomographic and microscopy-based far and near infrared fluorescence (NIRF, >650 nm) imaging methods offer high resolution, sensitivity, and depth penetration, there has been a lack of optimized NIRF agents to label and track cells in their native environments at different scales. In this study we labeled mammalian leukocytes with VivoTag 680 (VT680), an amine reactive N-hydroxysuccinimide (NHS) ester of a (benz) indolium-derived far red fluorescent probe. We show that VT680 diffuses into leukocytes within minutes, covalently binds to cellular components, remains internalized for days in vitro and in vivo, and does not transfer fluorescence to adjacent cells. It is biocompatible, keeps cells fully functional, and fluoresces at high intensities. In a tumor model of cytotoxic T lymphocyte (CTL) immunotherapy, we track and quantify VT680-labeled cells longitudinally at the whole-body level with fluorescence-mediated molecular tomography (FMT), within tissues at single cell resolutions by multiphoton and confocal intravital microscopy, and ex vivo by flow cytometry. Thus, this approach is suitable to monitor cells at multiple resolutions in real time in their native environments by NIR-based fluorescence imaging.

## Introduction

The growing appreciation that cellular and molecular mechanisms are controlled by the microenvironment in which they operate–in vitro models seldom recapitulate in vivo behavior–indicates a need to simultaneously monitor biological processes at various scales non-invasively. Optical imaging now allows in vivo visualization of biology as it unfolds, with relatively little perturbation of the native environment, but multi-scopic imaging that integrates single-cell and whole-body information from the same animal is often not possible, partly because of inadequate reporter tags. Most fluorescent cell tracker dyes emit at wavelengths under 650 nm (CFSE, CMTPX, BODIPY 630, DDAO-SE), where light is efficiently absorbed and scattered, thus limiting meso/macro-scopic analysis. Moreover, tissue autofluorescence at visible wavelengths confounds discrimination between target and background signal and is much lower in the NIR [1]. While design of cell trackers in the far and near-infrared region of the light spectrum partly resolves these problems, cell trackers must also be considered for multiphoton excitation, biocompatibility, and cellular retention if they are to report effectively on biology. Their optimization, therefore, remains a challenge for multi-scopic imaging.

Optical methods in current use include epifluorescence, confocal and multiphoton (MP) intravital microscopy (IVM) and fluorescence-mediated molecular tomography (FMT). For analysis at the mesoscopic level, FMT permits longitudinal assessment of fluorochrome concentrations in the whole body of mouse models [2]. Measurements are inherently quantitative and three-dimensional and the method has been used to report on enzyme activity, inflammation, and phagocytosis [1], [2]. At the microscopic level, IVM permits the dynamic study in situ of cell migration and cell-cell interactions in living animals in three dimensions [3]–[5]. Recent studies have used MP-IVM to explore the migratory and interactive activities of immune cells with their environment [3], [6]–[11]. Harnessing the capacity of these technologies to provide insight, in a single mouse with one reporter, into how cell-cell interactions influence and are influenced by their larger environment, is an unmet need.

In this study, we evaluated whether VT680 (peak excitation 670±5 nm, peak emission 688±5 nm), a near-infrared fluorochrome NHS ester, can be used as a multi-scopic cell tracker for in vivo optical imaging; we compared it to CFSE, a cell tracker currently available and in wide use at the microscopic level. We tested the capacity of VT680 to label and remain in cells, to keep cells alive and functional, and to report on biodistribution and T cell behavior in a tumor model of adoptive transfer immunotherapy in vivo using FMT and IVM, and ex vivo by flow cytometry.

## Results

### VT680 efficiently labels cells, remains internalized in vitro and in vivo, and does not interfere with cell function

**Figure 1:**
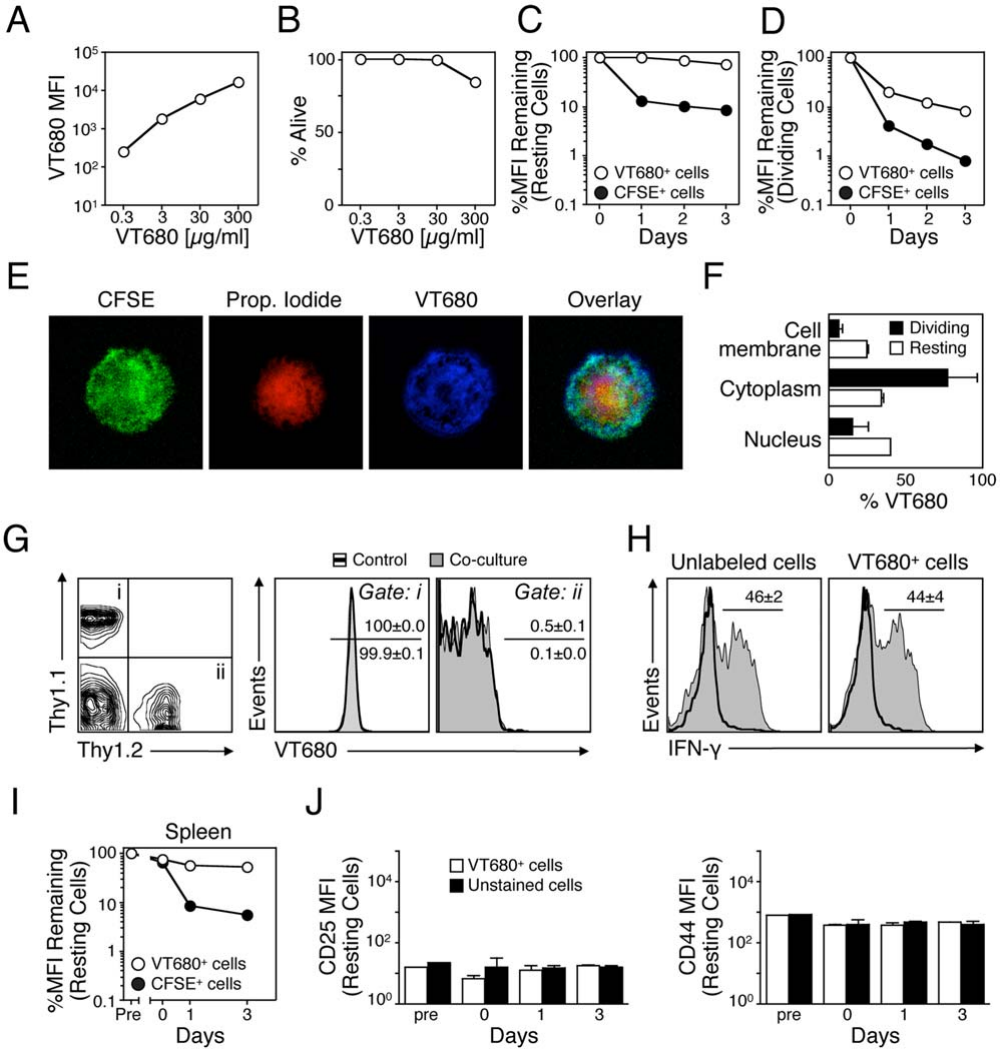
VT680 efficiently labels splenocytes, remains internalized in vitro and in vivo, and does not interfere with cell function. A. Splenocytes were incubated with increasing doses of VT680 and mean fluorescent intensity (MFI) for each dose is shown. B. Viability of splenocytes after labeling with increasing doses of VT680. C. In vitro retention of VT680 and CFSE in resting cells for three days after labeling (log scale). D. In vitro retention of VT680 and CFSE in dividing cells for three days after labeling (log scale). E. Cellular distribution of VT680 assessed microscopically. F. Cellular distribution of VT680 in resting and dividing cells assessed with a bio-cellular assay. G. VT680-labeled Thy1.1 cells were mixed in a 1∶1 ratio with unlabeled Thy1.2 cells and cultured together. A different group was cultured separately. 24 h later VT680 retention was assessed in Thy1.1^+^ (Gate i) and Thy1.2^+^ (Gate ii) cells. H. Expression of IFN-γ on naïve cells (thick-line histogram) and cells stimulated with PMA/Ionomycin (shaded histogram) that were either unlabeled or labeled with VT680. I. Retention of VT680 in vivo. Thy1.2 splenocytes were labeled with VT680 or CFSE ex vivo and injected to Thy1.1 mice. At indicated times recipient spleens (Thy1.1) were harvested and retention of label on donor cells (Thy1.2) was calculated. J. CD25 and CD44 expression. Thy1.2 splenocytes were labeled with VT680 and injected to Thy1.1 mice. At indicated times recipient spleens were harvested and CD25 and CD44 expression was measured on VT680-labeled, Thy1.2 donor splenocytes and unlabeled, Thy1.1 cells of recipient mice. Mean and SEM are shown, n = 3–5.

To test the cell-labeling capacity of VT680 we used readily abundant mouse splenocytes as a first model. Freshly isolated splenocytes were incubated for 30 min at 37°C with increasing doses of the fluorochrome. Mean fluorescent intensity (MFI), as determined by flow cytometry (FCM) (Laser Ex: 635; Em filters: 685/LP, 695/40), showed that VT680 tagged cells efficiently; increasing doses corresponded to increasing MFI and reached 4×10^4^ with 300 µg/mL compared to <10 MFI for control unlabeled cells (Fig. 1A). Concurrent analysis of cell survival immediately after labeling showed that VT680 was not toxic at doses up to 30 µg/mL (Fig. 1B). For all subsequent experiments, cells were labeled with 30 µg/mL, thus ensuring high MFI (1×10^4^–1.3×10^4^) and high viability (>95%). To assess fluorochrome retention, we investigated kinetics of VT680 release from resting (Fig. 1C) or dividing cells (Fig. 1D), daily for three days after labeling. In parallel, cells were stained with CFSE (5 µM), a gold standard tracer for ex vivo cell tracking [12]. When compared to MFI obtained immediately after labeling, VT680 fluorescence of resting cells was close to 100% during three days of culture (Fig. 1C). In contrast, 1 log of fluorescence was lost within the first 24 h when cells were labeled with CFSE, and continued to decrease for the next two days at a slower rate, albeit at a rate slightly higher compared to VT680 (−0.19 log/day for CFSE vs −0.06 log/day for VT680). The large loss of CFSE shortly after labeling may reflect CFSE staining a compartment that is rapidly exchanged, and the loss may indeed occur at times earlier than 24 h. VT680 retention, therefore, was at least as good as, if not better than, CFSE. Labeling did not alter survival of the cultured cells (data not shown). Similarly, in dividing cells, fluorescence loss was higher for CFSE than for VT680 (Fig. 1D). However, fluorescence in dividing cells was not characterized by discrete peaks as observed for CFSE, but rather a streak. It is possible this is a function of detecting fluorescence in the far-red range. Of note, injection of VT680 in vivo efficiently labeled circulating and splenic leukocytes but this untargeted agent was not selective for any cell type (data not shown).

To further determine the cellular distribution of VT680, labeled cells were fixed and stained with CFSE and propidium iodide (PI). As expected, CFSE, which binds amine groups, labeled the cytoplasm abundantly, while PI targeted the nucleus. Confocal microscopy analysis showed heterogeneous labeling by VT680 in the cell membrane, cytoplasm, and nucleus (Fig 1E). This heterogeneous labeling may also explain why discrete peaks were not observed for proliferating cells; subsequent cell division would not lead to equal distribution of the dye among daughter cells. Quantification of the relative distribution of VT680 involved a bio-cellular assay in which cellular compartments were extracted and analyzed separately. By this method, VT680 was shown to accumulate in the cell membrane, cytoplasm, and nucleus of both resting and dividing cells (Fig 1F). VT680 uptake by dividing T cells compared to resting cells was higher in the cytoplasm (80% vs 35%), but lower in the cell membrane (8% vs 25%) and nucleus (12% vs 40%), likely because the cytoplasm of cells selectively increases in size upon activation.

To investigate whether labeled cells can transfer VT680 to unlabeled neighboring cells, we co-cultured VT680-labeled Thy1.1 T cells with unlabeled Thy1.2 T cells (Fig 1G). All Thy1.1 T cells fluoresced at the wavelength indicative of VT680 while none of the Thy1.2 T cells showed VT680 fluorescence above background, demonstrating that VT680 did not transfer to adjacent cells. Moreover, VT680-labeled T cells retained their capacity to produce cytokines upon stimulation with PMA and Ionomycin, as evidenced by similar IFN-γ secretion (Fig. 1H).

We next analyzed the capacity of VT680-labeled cells to retain the fluorochrome in vivo. Thy1.2 splenocytes labeled ex vivo with either VT680 or CFSE were injected i.v. into Thy1.1 mice. Mice were euthanized at days 0 (i.e. ∼10 minutes), 1 and 3 d after injection, spleen was harvested, and donor cells were identified with anti-Thy1.2 antibody. Mean fluorescent intensity after 3 days neared ∼100% of the original label, while the MFI of CFSE-labeled cells decreased to 10% (Fig. 1I), a finding in line with our previous in vitro observations (Fig. 1C). In parallel, to test whether VT680 activates cells in vivo, we measured levels of CD25 and CD44 expression on Thy1.2 (VT680^+^) and Thy1.1 (unstained) cells (Fig. 1J). We found similar expression of both receptors at all time points, suggesting that VT680 does not induce T cell activation in vivo.

### VT680-labeled cytotoxic T lymphocytes (CTLs) remain functional and can be tracked individually in vivo

**Figure 2:**
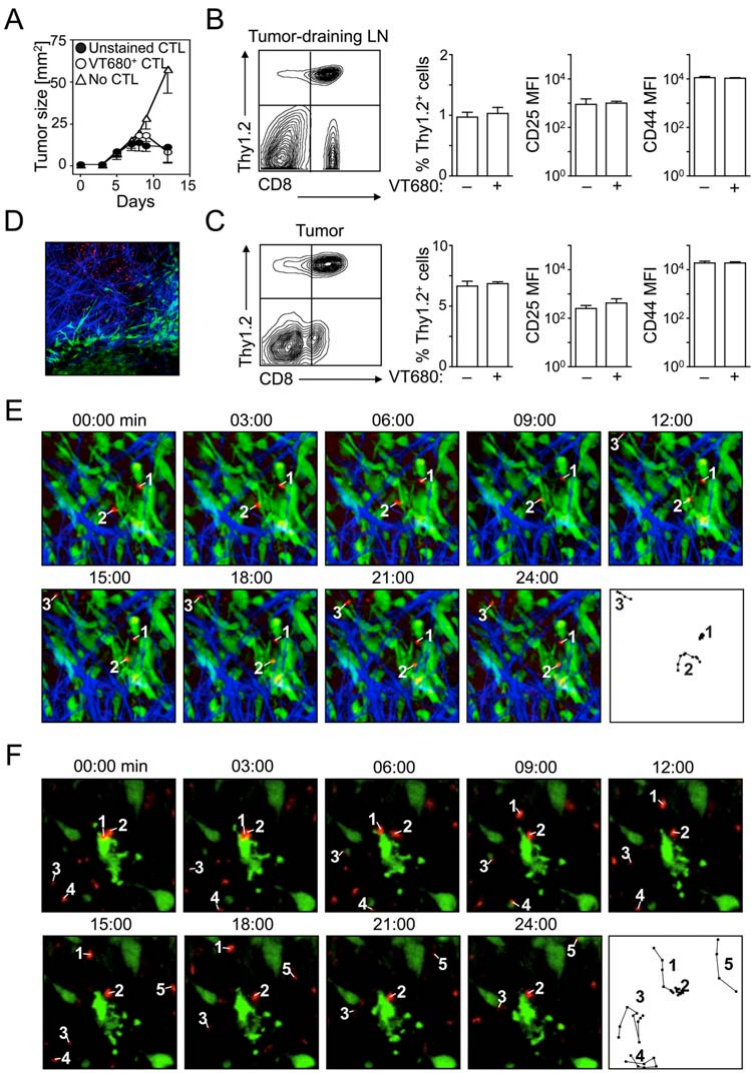
VT680-labeled cytotoxic T lymphocytes (CTLs) remain functional and can be tracked individually in vivo. CT44 tumors were injected s.c. into hind paws of Thy1.1 mice. After 7 days, activated Thy1.2 HA-specific CTLs either labeled with VT680 or unlabeled were injected i.v. A. Tumor growth kinetics in mice that did not receive CTLs and in mice that received VT680 and unlabeled CTLs. B. Relative distribution and activation status of VT680-labeled and unlabeled donor Thy1.2 CTLs retrieved from the tumor-draining lymph nodes. C. Relative distribution and activation status of VT680-labeled and unlabeled donor Thy1.2 CTLs retrieved from the tumor. Mean and SEM are shown, n = 3. D. Overview of tumor bulk. E. Multiphoton microscopy on day 2 after adoptive transfer. Paths of cell migration are shown in the last panel. F. Intravital microscopy on day 4. T cells (red) are in close contact to a cancer cell (green), which shows phenotypic changes suggestive of ongoing apoptosis. Paths of cell migration are shown in the last panel.

The ability of VT680 to prominently and stably label cells in vitro and in vivo suggests that the fluorochrome may be used as a reporter for cell migration and function in disease models. To test this, we employed a well-characterized tumor model of adoptive transfer immunotherapy. Tumors expressing a model Hemagglutinin (HA)-antigen were implanted s.c. in the hind paw and their growth was monitored for the duration of the experiment (Fig. 2A). In parallel, we expanded HA–specific CTLs in vitro. We obtained these cells from *TCR-CL4 RAG^−/−^* Thy1.2 BALB/c mice that express a transgenic TCR specific for the K_d_–restricted HA_512-520_ peptide. When stimulated in vitro with cognate peptide and IL-2, HA-specific CTLs kill HA^+^ CT44 tumor cells efficiently and selectively [13], [14]. Seven days after tumor implantation, Thy1.2^+^, HA-specific CD8^+^ cytotoxic T lymphocytes (CTLs) were tagged in vitro with VT680 and injected i.v. into Thy1.1^+^ tumor-bearing mice. Unlabeled CTLs served as controls. VT680-labeled CTLs were as efficient at controlling tumor growth as unlabeled CTLs (Fig. 2A), demonstrating that the imaging agent did not alter the CTL response in vivo. Five days after CTL injection, mice were euthanized and donor Thy1.2^+^ cells retrieved from the tumor-draining lymph nodes (Fig. 2B) and tumor (Fig. 2C). Retrieved numbers of Thy1.2^+^ CTLs were similar irrespective of VT680 labeling. Consistent with in vitro observations (Fig. 1D & G), Thy1.2^+^ CTLs labeled with VT680 remained fluorescent, did not transfer fluorescence to adjacent cells, and showed the same level of CD25 and CD44 expression when compared to unlabeled CTLs, further substantiating the conclusion that VT680 does not alter cell function.

We next tested whether immune cells labeled with VT680 can be detected by intravital microscopy. To this end, we injected 10^7^ VT680-labeled HA-specific CTLs i.v. into mice with GFP+ HA-expressing CT44 tumors growing in skin-fold chambers. We found that both confocal (excitation: 637 nm, emission: 660/LP) and multiphoton (excitation: 820 nm, emission: 560/LP, 620/100 nm BP) microscopy allowed detection of the CTL in tumor-associated stroma. Around two days after adoptive transfer, CTLs accumulated at the periphery of the bulk of tumor cells, (Fig 2D and Movie S1) while only few CTLs were found inside the tumor (Fig 2E). Around four days after adoptive transfer, more CTLs had accumulated inside the tumors (Fig 2F). In line with two recent reports [15], [16], CTLs in stroma of regressing tumors initially interacted for prolonged periods of time with tumors and thus displayed relatively low velocity/motility. Disappearance of tumor cells at later time points coincided with the detection of CTL that now showed higher velocity/motility and that did not interact with tumor cells, at least during the 30–60 min recordings (Fig 2 E & F). Of note, we observed that tumor cells could be simultaneously attacked by two CTLs (see Fig 2F, cells #1 & 2 and Movie S2), which is in contrast with our previous observations in which CTL attack of antigen-pulsed B cells was always characterized by monogamous interactions [10]. Here we show that VT680 can be used for intravital microscopy and thus may be useful in future experiments in combination with other markers for multicolor imaging to simultaneously track multiple cell types. The dye became highly compartmentalized in T cells, appearing as bright puncta in the uropod of migrating cells. Moreover, we found that VT680-labeled CTLs could be detected for up to 6 days after injection, thus allowing the investigator to study the unfolding of immune responses over rather large periods of time.

### VT680-labeled CTL biodistribution can be quantified and tracked non-invasively in vivo

**Figure 3:**
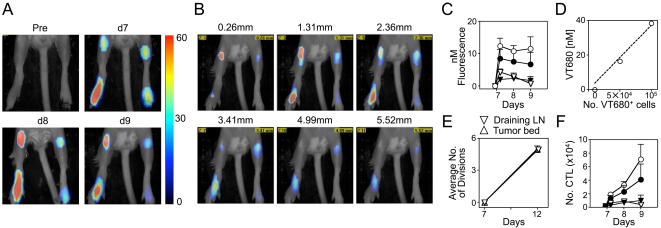
VT680-labeled CTL accumulation can be quantified and tracked mesoscopically in vivo. CT44 tumor cells were injected s.c. in the right hind paws. After 7 days of growth VT680-labeled HA-specific CTLs were injected. A. Fluorescent-mediated molecular tomography (FMT) depicting kinetics of CTL accumulation in tumor and lymph nodes. B. Z stacks of FMT images on day 8. C. Correlation of FMT signal vs days after CTL transfer in tumors (white) and draining lymph nodes (black) from the right, tumor-bearing, paw (circles) and the left, contra-lateral, paw (triangles). D. Number of cells vs. fluorescence determined by phantom experiments. E. Average number of CTL cell divisions during 5 d after injection determined by VT680 fluorescence loss. F. Estimation of CTL accumulation in tumors (white) and draining lymph nodes (black) from the right, tumor-bearing, paw (circles) and the left, contra-lateral, paw (triangles), determined by combining information from D and E.

Microscopy studies with VT680-labeled tumor-reactive CTLs permit visualization of efficient killing at the single-cell level, but are insufficient at determining the biodistribution and number of tumor reactive CTLs accumulating at the stroma. Complementing these studies with a quantitative mesoscopic modality such as fluorescent-mediated molecular tomography (FMT) would provide further insight into the specificity, scope, and magnitude of the response. As VT680 emits at a NIR wavelength where autofluorescence and tissue absorbance are minimal, we hypothesized that the agent's optical properties will allow concurrent mesoscopic evaluation of CTL activity. HA-bearing tumors were implanted and allowed to grow. Six days later, VT680-labeled HA-specific CTLs were injected. Mice were imaged before CTL injection and on three consecutive days after injection. Regions of interest (ROI) were generated around the tumor and the draining lymph node as well as on the corresponding locations on the contralateral leg. VT680 signal was elevated in HA-bearing tumors for the duration of the experiment (Fig 3A). Fluorescence appeared within the tumor one day after CTL transfer and remained high for several days throughout the tumor bulk, as assessed by slices through the Z plane (Fig. 3B and Movie S3). Correspondingly, fluorescence in the tumor-draining lymph nodes was higher in legs implanted with tumors compared to the contra-lateral leg. In contrast, low fluorescence was observed in the contra-lateral leg and the corresponding lymph node, indicating migration of some HA-specific CTLs at sites not expressing HA. This was consistent with flow cytometric analysis (data not shown).

To establish whether FMT, a modality with the advantage of being inherently quantitative, can be used to predict the number of cells at the tumor site, several parameters were considered. First, fluorescence signal, quantified in nM fluorescence units (Fig 3C), showed more pronounced fluorescence in tumor-bearing paws and tumor-draining lymph nodes. So as to determine the relationship between nM and number of cells, phantom experiments were conducted with known cell number implanted s.c. (Fig 3D). Average number of cell divisions, as occurs in vivo, could be calculated because VT680 does not lose fluorescence in resting cells (Fig 1C & D and Fig 3E). By taking these variables into account, it was determined that ∼7×10^4^ CTLs were present at the tumor 3 days after injection and ∼4×10^4^ CTLs could be found in the draining lymph nodes. This was consistent with the number of cells retrieved from lymph nodes and determined with the use of flow cytometry (4–10×10^4^). Together, these data show that biodistribution can be quantified non-invasively in vivo over time and that CTLs continue to accumulate in the tumors and lymph nodes during the course of the experiment.

## Discussion

In this study, we show that VT680, a near-infrared fluorochrome NHS ester, allows tracking of cells with multiple in vivo imaging modalities at different scales. FMT monitors distribution of cells in the entire animal while IVM and MP-IVM allow for zooming into specific regions of interest for analysis of activity at the single cell level. The agent diffuses into cells within minutes, internalizes in various cellular compartments, is fully biocompatible, remains internalized for days in vitro and in vivo, and does not transfer fluorescence to adjacent cells. In comparison, cells labeled with widely used dyes such as CFSE lose on average one order of magnitude of fluorescence within 24 hours.

Cells in their native environment can behave differently compared to cells that are placed in culture [10]. In vivo, cell behavior is influenced by multiple other factors such as complex anatomical features, forces of fluid flow, the extracellular matrix, as well as adhesion with neighboring cells and mixtures of cytokines. Understanding the activity and function of immune cells and, more broadly, dissecting the complexity of biological systems, necessitates development of in vivo imaging technologies that are robust and quantitative. Cell labeling agents already available for optical imaging such as GFP, RFP, CFSE, and CMTMR are useful for microscopic applications but are less suitable for mesoscopic analysis as they offer limited tissue penetration. VT680 penetrates tissue because hemoglobin, water, and lipids have their lowest absorption coefficients in the NIR region of 650–900 nm. As a result, VT680 may be used not only for ex vivo flow cytometric analysis but also for several in vivo imaging modalities that track accumulation both mesoscopically and microscopically. The combination of micro and mesoscopic imaging in the same animal offers several advantages because it allows the simultaneous evaluation of cellular activity and biodistribution. Such advances will foster a systems approach in which activities at the cell level are evaluated in the context of the entire animal.

## Materials and Methods

### Animals

Female Balb/c (Thy1.2^+^) mice were purchased from Taconic farms (Germantown, NY, USA). *TCR-CL4* RAG^−/−^ BALB/c mice expressing a TCR specific for K^d^/HA_512-520_ were generated as described [13]. DUC18 BALB/c mice expressing a TCR specific for K^d^/tERK-I _136-144_ [17] and Thy1.1 BALB/c mice were obtained from Dr. Paul Allen and bred in-house. All animals were housed in a specific pathogen-free environment and used at 8 to 15 weeks of age. The institutional subcommittee on research animal care approved all animal studies.

### Cells

Single cell suspensions were prepared by homogenizing the spleen (Potter™ homogenizer) and filtering single-cell suspensions through 70 µm cell strainers. Cells were washed twice and resuspended in complete medium for further experiments. The following complete medium was used: 86% vol. RPMI 1640, 1 mM sodium pyruvate, 10 mM HEPES, 2 mM glutamine, 1% penicillin/streptomycin, 50 µM mercaptoethanol, 10% FCS. To stimulate splenocyte proliferation, 4×10^6^ cells were cultured in 1 ml complete medium for 3 days supplemented with purified anti-CD28, 37.51 (2 µg/ml, BD BioSciences) and recombinant (r)IL-2 (20 ng/ml, R&D Systems) in 24-well plates that had been previously coated for 1 h at 37°C with purified anti-CD3, 145-2C11 (1.5 µg/well, BD Biosciences). To stimulate CL4 cell proliferation, cells were cultured in complete medium with 1 µg/mL HA_512-520_ peptide for 1 h, washed and maintained at 37°C. Starting on day 2 and every second day, cells were harvested and incubated in fresh complete medium supplemented with 20 ng/ml rhIL-2 (R&D Systems) until day 7.

### Cell labeling

VivoTag 680 (VT680; Ex: 670±5 nm, Em: 688±5 nm; MW: 1240 gmol^−1^) was commercially obtained from VisEn Medical (Woburn, MA), dissolved in DMSO and stored in aliquots of 10 mg/ml at −20°C. Sufficient volume to ensure a final VT680 concentration of 0.3, 3, 30 or 300 µg/ml was added to 4×10^6^ cells/ml previously resuspended in complete medium at 37°C. A dose of 30 µg/ml corresponds to 24.1 µM. Cells were incubated with VT680 at 37°C for 30 min at 5% CO_2_. The cells were washed twice after labeling. Preliminary experiments showed that presence of serum does not impair cell labeling. Cells were incubated in the presence of different doses of VT680 for 30 min at 37°C. For CFSE labeling, cells were incubated in 5 µM CFSE in RPMI for 15 minutes at 37°C. Cell viability was assessed microscopically with Trypan Blue exclusion (Cellgro Mediatech, Inc.) and further corroborated with the forward scatter vs. side scatter profile. For intracellular dye localization, cells were first labeled with VT680, fixed with Cytofix/Cytoperm™ kit (BD Biosciences, USA), then labeled with PI (propidium iodide) (1/1000) to identify the nucleus. Cells were prepared on slides by cytocentrifugation (Shandon, Inc.) at 10×g for 2 min (Shandon, USA). Ex vivo cell observations were made on a Zeiss Axiovert 200 microscope with fluorescence and bright-field capabilities. For cell fractioning, the subcellular proteome extraction kit (ProteoExtract®, CalbioChem, USA) was used according to the manufacturer's protocol. Briefly, VT680 labeled splenocytes were successively sedimented three times. Owing to different extraction buffers, we collected four different fractions containing the cytosol, the membrane protein extract, proteins from the nucleus, and proteins of the cytoskeletal matrix. VT680 was quantified by measuring the near infrared fluorescence (Excitation: 670±15; Emission: 700±15) of each fraction in a photometer microplate reader (safire2, TECAN, Durham NC, USA). Amount of VT680 in each fraction was referenced to a standard curve (increasing concentration of VT680 diluted in PBS).

### Flow cytometry

Cells were incubated with the following mAbs: Thy1.2 (CD90.2)-PE, 53-2.1; Thy1.1 (CD90.1)-FITC, OX-7; IFN-γ- FITC, XMG1.2; CD25-FITC, clone; CD44-APC, IM7; CD8-PerCP, 53-6.7. For intracellular staining of IFN-γ cells were stimulated for 5 h with 1 µg/ml phorbol 12-myristate 13-acetate (PMA) and 0.25 µg/ml Ionomycin and in the presence of 10 µg/ml Brefeldin A after 1 h (Sigma-Aldrich) and permeabilized and fixed with a Cytofix/Cytoperm Kit (BD Biosciences). Data were acquired on an LSRII (BD Biosciences). To detect VT680, a red Helium-Neon 635 nm laser was used for excitation and a 685/LP and 695/40 filter configuration was used for detection.

### Adoptive transfer and tumor model

The tumor cell line CT44 was generated by transfecting CT26 cells (a cell line derived from a chemically induced murine colon carcinoma) with a fusion protein of influenza hemagglutinin and EGFP [13]. Anesthetized animals received 10^6^ tumor cells in 50 µL PBS s.c. into the upper side of the hind paw. 10^7^ VT680 or CFSE-labeled Thy1.2 splenocytes were injected intravenously. Tumor size was monitored on a daily basis using a caliper.

### Confocal and multiphoton microscopy

In vivo monitoring of cell migration was performed in the dorsal skin fold chamber model, as previously described [18]. On day zero, 10^6^ tumor cells were injected i.m. in the center of the window. After 6 days, VT680-labeled CL4 cells were injected i.v. Confocal and multiphoton microscopy experiments were performed on days 7 to 10. For laser scanning microscopy (Olympus IV100), VT680 was excited with a red laser diode of 637 nm and detected with a 660/LP filter. For multiphoton microscopy (on a BioRad Radiance 2100 MP centered around an Olympus BX51 equipped with a 20×/0.95 NA objective lens), the dye was excited with a pulsed Ti:Sapphire laser (MaiTai HP, Spectra-Physics) tuned to 820 nm. This wavelength was chosen because VT680 has a peak in its two-photon cross-section at 820 nm. Since our available detection optics were suboptimal for collection of VT680 emission, which is maximal at 688 nm, we prioritized optimal excitation of VT680 over that of eGFP, for which we otherwise found 920 nm to be optimal. Although excitation of eGFP is suboptimal at 820 nm, visualization of tumor cells was still possible due to their high expression of eGFP. A 560 nm DCLP filter and a 620/100 nm bandpass filter were used for the non-descanned detection of VT680, while EGFP emission and second harmonic signals from collagen were recorded using 515/30 nm and 400/40 nm bandbass filters, respectively. For four-dimensional recordings of T cell migration in tumors, stacks of 11 optical sections were acquired every 30 seconds for 30 minutes with an optical zoom of 3× to provide image volumes of 40 µm in depth and 208 µm in width and length. Sequences of image stacks were transformed into volume-rendered, four-dimensional movies using Volocity software (Improvision, Coventry, UK) and exported as Quicktime movies. Motion-artifacts in recordings were corrected using the auto-alignment plugin (stackreg) of ImageJ (http://rsb.info.nih.gov/ij/).

### FMT

Three to five days prior to imaging mice were placed on a low manganese diet (Harlan, Indianapolis IN) to reduce autofluorescence caused by normal mouse chow when imaging. The imaging site (hind paws and legs up to the knee joint) was shaved and depilated to remove all hair, which is a source of interference with fluorescent imaging. Mice were imaged on the FMT system (VisEn Medical) before injection and on days 1–4 following i.v. injection of labeled cells. Mice were anesthetized by inhalation of isoflurane, and placed on the imaging cartridge. Anesthesia was maintained by the use of a nose cone, and mice were placed within the imaging chamber. Reflectance images were taken in white light and fluorescent modes. The imaging chamber was filled with an index matching solution (VisEn Medical) as per the vendors instructions. Non-invasive fluorescent tomographic imaging was carried out in the VT680 channel. After imaging, the chamber was drained, the mouse removed, and allowed to recover. The FMT software allows for the 3D reconstructions of the imaging data utilizing a normalized Born equation. Following the reconstruction, volumes of interest (VOI's) were selected by drawing regions of interest (ROI's) in all 3 imaging planes (X, Y, Z). A mean fluorescent value and total VOI volume and fluorochrome concentration were generated for VOI's encompassing the hind paws and the draining lymph nodes. FMT phantom experiments were performed to generate standard curves and calibrate the FMT system for the VT680 fluorochrome. Phantoms consisted of pure VT680 dye diluted into PBS at various concentrations and VT680-labeled cell suspensions in PBS.

## Supporting Information

Movie S1 Multiphoton intravital microscopy of tumor bulk on day 2 after T cell transfer. Tumor cells (green) are in contact with VT680-labeled T cells (red). Collagen fibers (blue) are seen.
(0.49 MB MOV)

Movie S2 Confocal intravital microscopy of the tumor bulk on day 4 after T cell transfer. VT680-labeled T cells (red) engage a cancer cell (green).
(0.52 MB MOV)

Movie S3 Fluorescence-mediated molecular tomography on day 1 after VT680-labeled T cell transfer. Fluorescence is seen through Z stacks at the tumor and the tumor draining lymph node.
(0.03 MB MOV)
